# Botulinum Neurotoxin Heavy Chain Belt as an Intramolecular Chaperone for the Light Chain

**DOI:** 10.1371/journal.ppat.0030113

**Published:** 2007-09-28

**Authors:** Axel T Brunger, Mark A Breidenbach, Rongsheng Jin, Audrey Fischer, Jose S Santos, Mauricio Montal

**Affiliations:** The Scripps Research Institute, United States of America

## Background

Botulism is a neuroparalytic illness caused by botulinum neurotoxin (BoNT). Seven BoNT serotypes (designated as A to G) are produced by *Clostridium botulinum,* a spore-forming, obligate anaerobic bacterium. BoNT, widely considered the most potent toxin known and a major bioweapon [1], is a potent blocker of synaptic transmission in peripheral cholinergic nervous system synapses, thereby causing paralysis. Based on its exquisitely powerful neuroparalytic activity, BoNT has gained tremendous popularity in the past few years since becoming the first biological toxin (BoNT serotype A) to receive US Food and Drug Administration approval for the treatment of human disease [2].

**Figure 1:**
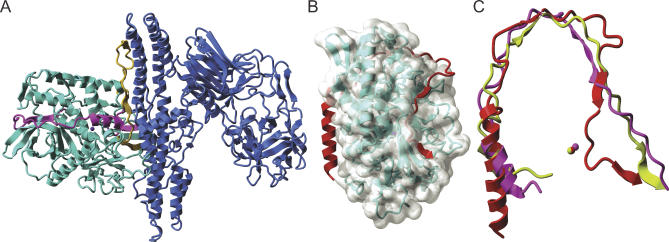
Structures of BoNT/A Holotoxin, BoNT/A LC-sn2 Complex, and Overlay of sn2 Segment with Belt Regions of BoNT/A and BoNT/B (A) Structure of BoNT/A. The Cα backbone of the LC (left) is represented as cyan ribbons; the purple sphere highlights the catalytic Zn^2+^ at the protease active site. The HC belt segment encompassing residues 492–545 is displayed in magenta and the 449–491 region in gold. The HC is depicted in blue, in which the helical module (middle) is the translocation domain, and the two sub-domains consisting primarily of β-strands constitute the receptor-binding domain (right). (B) Structure of BoNT/A-LC in complex with the sn2 segment of SNAP-25 [16]. The Cα backbone of the LC is represented as cyan ribbons and its molecular surface in transparent grey. The sn2 segment is depicted in red and the catalytic Zn^2+^ at the active site as a purple sphere. (C) Superposition of the structures of the sn2 segment in complex with the LC/A, the HC belt of BoNT/A, and the HC belt of BoNT/B. LC removed for display. For the superposition, the backbone atoms of the LCs were used for the best fit between the structures. sn2 segment depicted in red; overlay of the Cα backbone of BoNT/A [4] and BoNT/B [6] belts represented as magenta and lime ribbons. Spheres represent the catalytic Zn^2+^ at the active site of BoNT/A (red and magenta) and BoNT/B (lime). All images were rendered with YASARA [40].

Biochemically, BoNTs are synthesized as single polypeptide chains and then cleaved by bacterial proteases into a di-chain molecule linked by a disulfide bond: an ∼50-kDa light chain (LC) and an ∼100-kDa heavy chain (HC). Structurally, BoNTs encompass three modules [3–6]: the N-terminal LC is a metalloprotease, whereas the HC comprises the translocation domain (the N-terminal segment) and the receptor-binding domain (the C-terminal segment). The modular architecture of the neurotoxin is clearly visible in the crystal structures of BoNT/A [4] (Figure 1A) and BoNT/B [6]. All seven BoNT serotypes exhibit significant amino acid sequence conservation [5], although all are antigenically distinct.

It is generally agreed that BoNTs exert their neurotoxic effect by a four-step mechanism [3,7] that involves (1) binding to high-affinity receptors on peripheral nerve endings, (2) receptor-mediated endocytosis, (3) LC translocation across endosomal membranes into the cytosol upon exposure to endosomal pH, and (4) proteolytic degradation of target. The BoNT LCs are sequence-specific endopeptidases that cleave SNARE (soluble N-ethylmaleimide-sensitive factor attachment protein receptor) proteins. SNAREs form a complex that mediates synaptic vesicle fusion [8–10]. Accordingly, SNARE proteolysis destabilizes or prevents full assembly of the SNARE core complex, abrogating fusion of synaptic vesicles with the plasma membrane, thereby aborting neurotransmitter release [3,11]. BoNT serotypes A, E, and C all cleave the plasma membrane–associated protein SNAP-25 (synaptosome-associated protein of 25 kDa), and serotype C also cleaves the plasma membrane–associated SNARE syntaxin. In contrast, BoNT/B, D, F, and G all proteolyze synaptobrevin, a synaptic vesicle–associated membrane protein, also known as VAMP, at unique sites [3]. The active site region of the BoNT LCs shares structural similarity to the Zn^2+^-metalloprotease thermolysin [4,6,12–17]. In contrast to other Zn^2+^-proteases, the BoNTs require an extended enzyme-substrate interface for optimal catalytic efficiency [18–20]. Indeed, the X-ray structure of BoNT/A-LC in complex with sn2 [16]—the C-terminal residues 141–204 of BoNT/A substrate SNAP-25—revealed an extensive array of substrate binding sites distant from the active site (exosites) that orient the substrate onto the vicinity of the active site and determine the target specificity [16,21].

A key step for intoxication is the translocation of endocytosed toxin across intracellular membranes to reach its cytosolic targets [3]. The HC likely acts as both a channel and a transmembrane chaperone for the LC protease to ensure a translocation-competent conformation during transit from acidic endosomes into the cytosol [22–24]. The details of the translocation process are largely unknown. However, available crystal structures of BoNT/A [4] and BoNT/B [6] holotoxins and of BoNT/A-LC in complex with sn2 [16] provide illuminating clues about possible mechanisms, which we consider next.

The so-called translocation domain belt is a most intriguing structural feature in the crystal structures of both BoNT/A [4] and BoNT/B [6]: It is a loop in a mostly extended conformation (consisting of residues 492–545 for BoNT/A, and 481–532 for BoNT/B) that wraps around the catalytic domain in the structures solved at pH 7.0 and 6.0, respectively. The active site of the LC is buried ∼20 Å deep in the protein and is accessible through a negatively charged crevice, which may be partially occluded by the belt in the unreduced holotoxin. The belt is highlighted in magenta on the structure of the holotoxin/A [4] displayed in Figure 1A. In addition, there is a second unstructured loop encompassing residues 449–491 for BoNT/A; this segment, depicted in gold, is partially apposed to the LC, perpendicular to the belt, and is parallel to the long helices of the translocation domain. The structure of the BoNT/A-LC complex with the sn2 segment [16] is shown in Figure 1B. Figure 1C displays a superposition of the Cα positions of the structures of the BoNT/A-LC complex with the sn2 segment (red), the HC belt of BoNT/A (magenta), and the HC belt of BoNT/B (lime). The belt of BoNT/A is more distant to the catalytic Zn^2+^ (distance between M^530^ and Zn^2+^ is ∼15 Å) than the cognate substrate (distance between Q^197^ and Zn^2+^ is ∼7 Å). Note the remarkable structural similarity between sn2 and the belt in the absence of stringent sequence similarity [5,6]. This is relevant given the low sequence similarity (24%) of the belts among the seven BoNT serotypes and the related clostridial toxin, tetanus [5,25].

What is the role of this belt?

## Hypothesis

We propose that the belt region of the BoNT HC is a surrogate pseudosubstrate inhibitor of the LC protease and acts as a chaperone during translocation across the endosomal membrane into the cytosol. The key points are: (1) The intrinsically unstructured sn2 fragment of SNAP-25 [16,26] adopts partial secondary structural elements upon binding to the LC in the binary complex crystal structure [16] and occupies a similar position as the belt in the holotoxin crystal structures of both BoNT/A [4] and BoNT/B [6]. (2) In analogy to other “intrinsically unstructured proteins” (IUPs) [27,28], the belt undergoes binding to its LC partner, thereby functioning as a chaperone [24]. (3) The belt occupies the exosites, the extensive enzyme surface allocated for substrate binding, yet it does not contain the scissile bond, thus potentially inhibiting the LC protease.

## Mechanism

**Figure 2:**
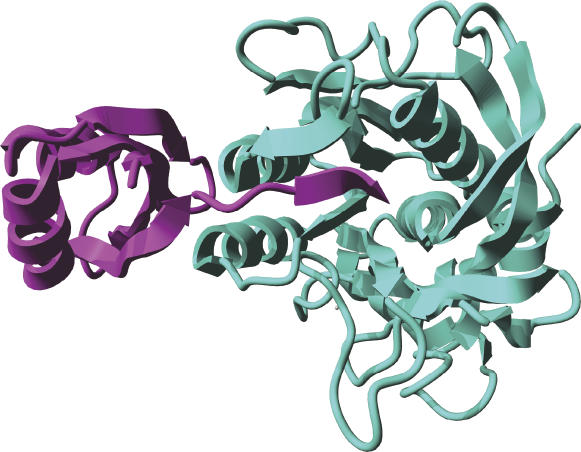
Structure of the Subtilisin–Pro-Domain Complex The Cα backbone of subtilisin is represented as cyan ribbons and that of the pro-domain in magenta. Note that the C-terminus of the pro-domain is lodged in a crevice at the protease active site; the tip of the β-strand highlights Y^77^ at the active site. Image rendered using subtilisin BPN' prosegment complexed with a mutant subtilisin BPN' [31,32] with YASARA [40].

A number of plausible mechanisms can be envisioned. One, protein-assisted unfolding and pseudosubstrate-assisted refolding of the protease could be an attribute of chaperone action. There is precedence for protease inhibitors acting as intramolecular chaperones [29,30]. A case in point is subtilisin, for which propeptides, located between the signal peptide and the mature segments of the protease, function as protease inhibitors by lodging into the substrate binding pocket [30]. These peptides are effectively IUPs [26] because they lack 3-D structure in isolation, yet adopt secondary structure upon forming a complex with the cognate protease [29–31]. They are involved in the last steps of protein folding of the enzyme. The crystal structure at 2.0 Å resolution of the propeptide–subtilisin complex shows that the prosegment C-terminus (Figure 2, magenta) binds in the enzyme active site (Figure 2, cyan) in a product-like manner with Y^77^ (tip of the β-strand) in the P1 binding pocket [32,33]. POIA1 (Pleurotus ostreatus proteinase A inhibitor 1, PDB accession code 1ITP [34]), a mushroom peptide that acts as an intramolecular chaperone and inhibitor to subtilisin yet has only 18% sequence similarity to the cognate propeptide, has a similar tertiary structure to that of the propeptide of subtilisin. Similar mechanisms have been found for other bacterial proteases, including metalloproteases [29]. The analogy that emerges from the considerations described here for BoNT is remarkable and may be the crucial event underlying the activity of the BoNT HC belt as both an inhibitor and chaperone. However, the belt does not actually protrude into the BoNT active site. The putative inhibitory activity of the belt would therefore be restricted to the remote substrate binding interfaces of the exosites.

Two, the belt as a continuous segment, residues 449–545 for BoNT/A, may undergo a concerted structural transition at endosomal pH with profound consequences for the translocation process [24]. It is conceivable that a pH-induced transition of this segment may trigger the insertion of the translocation domain into the membrane. Since the belt embraces the LC, the belt may be a structural entity that facilitates or coordinates the concerted partial unfolding of the LC at the endosomal acidic pH and directs the beginning of its translocation through the membrane.

Three, a surrogate pseudosubstrate role is plausible. There is weak sequence similarity between the HC belt and sn2, which extends up to residue D^195^ of SNAP-25 and E^528^ of the HC belt [16,21,35]. Furthermore, the LC undergoes autocatalytic proteolytic fragmentation [17,36], which is prevented in the presence of a competitive inhibitory peptide with a sequence of CRATKML [19]; this peptide closely emulates the sequence of the SNAP-25 C-terminal fragment released by proteolysis of SNAP-25 (residues 197–203 with sequence QRATKML [20], in which the scissile bond is between Q^197^ and R^198^). However, this non-specific autocatalytic activity is known to occur only at high enzyme concentrations, such as in the context of crystallization trials, so a physiological role is questionable.

It is conceivable that combinations of these three mechanisms act in concert to enhance translocation efficiency. A relevant example is the extensively studied bacterial α-lytic protease (α-LP) [37,38]. The native state of α-LP is unstable and, if unfolded, exhibits a large barrier to refold. Folding of α-LP requires the chaperone activity of its N-terminal pro-domain, which confers strong inhibition on the protease. α-LP initiates degradation of the pro-domain by proteolytic cleavage of an intervening loop at the C-end of the pro-domain, thereby releasing the α-LP from the pro-domain and allowing folding. Cleavage of α-LP pro-domain, therefore, enables efficient folding by lowering the free energy of the folded state and lowering the transition state barrier between unfolded and folded states. Upon release of the pro-domain, the kinetic barriers for unfolding dramatically increase and the α-LP becomes highly protease resistant. Concurrently, the folded state becomes destabilized yet remains kinetically trapped in its native state by a large transition state barrier. We speculate that the belt could enable efficient folding and/or unfolding akin to the α-LP pro-domain. Once it is released inside the neuron, the protease is kinetically trapped and resistant to degradation by cellular proteases or autoproteolysis, thereby implying a convergence of chaperone and surrogate substrate mechanisms. Combined with the exquisite neurotropism of BoNT conferred by its receptor-binding domain, and the target specificity and optimal catalytic efficiency endowed on its protease domain, the holotoxin emerges as a marvel of protein design.

## Concluding Remarks and Perspective

These hypotheses naturally lead to testable questions: Is a beltless holotoxin toxic in vivo, e.g., in the context of a mouse toxicity bio-assay [39]? Is the belt required for channel formation? Is it required for LC translocation? Is the belt the trigger for translocation or a modulator? Are there conformational transitions upon entering the acidic environment of the endosome? The implication is that the belt region of BoNT/A HC must be subjected to a rigorous structural and functional analysis to evaluate its possible role in the translocation process, in particular with regards to a pH-induced conformational change. Accordingly, beltless variants of the HC, in which the belt region is eliminated or systematically truncated, could be recombinantly expressed, and their channel and translocation activities examined after reconstitution in lipid bilayers [24] and in neuronal cells [22,23] as described for the intact BoNT/A.

A surrogate pseudosubstrate role of the belt could be probed by using synthetic peptides that mimic the amino acid sequence of the belt, yet incorporate SNAP-25 residues present at the toxin cleavage site as potential toxin substrates. Conversely, one could design a synthetic holotoxin in which the HC belt is replaced by the sn2 segment of SNAP-25 (Figure 1B), comprising a non-cleavable bond instead of the native scissile bond. Would this chimera exhibit translocation features comparable to those of the native holotoxin?

Are the belt regions truly IUPs, and do they contribute to the thermodynamic stability of the LCs? To assess whether belt peptides are true IUPs, their solution structures could be assessed with circular dichroism and nuclear magnetic resonance spectroscopies. To investigate the chaperone activity of the belt, the unfolding and refolding kinetics of LC protease could be studied in the absence and presence of peptides that imitate the belt (Figure 1C) and compared to those of a complex of LC/A and sn2 (Figure 1B). Answers to these questions could substantially improve our understanding of the most enigmatic step in the molecular mechanism of BoNT intoxication. 

## Supporting Information

### Accession Numbers

The Protein Data Bank (PDB, http://www.rcsb.org/pdb/) accession codes for the proteins discussed in this paper are BoNT/A (3BTA [4]), BoNT/A-LC in complex with the sn2 segment of SNAP-25 (1XTG [16]), BoNT/B (1EPW [6]), and subtilisin BPN' prosegment complexed with a mutant subtilisin BPN' (1SPB [32,33]).

## Abbreviations

α-LP: α-lytic protease
BoNT: botulinum neurotoxin
HC: heavy chain
IUP: intrinsically unstructured protein
LC: light chain
SNAP: synaptosome-associated protein
SNARE: soluble N-ethylmaleimide-sensitive factor attachment protein receptor
